# Hepatobiliary Ascariasis in a Piglet

**DOI:** 10.1007/s11686-024-00813-2

**Published:** 2024-02-29

**Authors:** Claudia Tamponi, Lia Cavallo, Giorgia Dessì, Francesco Sardu, Carlo Carta, Andrea Corda, Giovanni Pietro Burrai, Antonio Varcasia, Antonio Scala

**Affiliations:** 1 Department of Veterinary Medicine, University of Sassari, Via Vienna, 2, 07100 Sassari, Italy; 2 Local Health Unit, Oristano, Italy

**Keywords:** *Ascaris suum*, Pig, Bile ducts, Ultrasound, Parasitology

## Abstract

**Purpose:**

Ascariasis caused by the helminth *Ascaris suum* is the most common parasitosis of swine worldwide and it may involve all age categories of pigs. The present study reports an unusual localization of *A.* *suum* worms in the biliary system of a piglet slaughtered for human consumption.

**Methods:**

The liver was subjected to ultrasound scan and pathological examination. The isolated worms were morphologically examined and the DNA was extracted for the molecular identification of the species involved.

**Results:**

A total of 43 preadult nematodes were found within the gallbladder and the bile ducts. Parasites were morphologically identified as belonging to the genus *Ascaris* and molecularly as *A. suum*. At gross examination, the liver was moderately enlarged, with the bile ducts severely dilated. A chronic inflammatory infiltrate was noted, often centered around ectatic bile ducts (up to 5 mm in diameter), lined by hyperplastic epithelium and filled with sections of nematodes. The worm sections showed smooth cuticle, coelomyarian musculature, and an intestinal tract lined by columnar, uninucleated cells within a pseudocoelom. The ex vivo ultrasonographic examination of the liver allowed the visualization of several nematodes in the bile duct lumen and could be suggested for *in vivo* diagnosis. Unfortunately, the absence of the intestine did not allow to define the pathogenesis of the infection.

**Conclusion:**

Although, given the unusual nature of this finding, it is difficult to identify predisposing factors for this *A. suum* localization, it suggests that ascariasis should be considered in the differential diagnosis of pigs with hepatobiliary disease.

## Introduction

Ascariasis is the most common parasitosis in pigs worldwide and is mainly caused by the helminth *Ascaris suum,* which belongs to the Family Ascarididae and which can reach a relevant size, of up to 40 cm in females [1]. *Ascaris lumbricoides* can also grow in pigs and both parasite species can mature in humans [2].

This parasitosis has a global distribution and is detected in both organic [3, 4] and industrial pig farms, especially in fattening pigs and old sows [5–7]. *Ascaris* *suum* infection can affect all age categories of pigs and depends on several factors mainly related to the type of rearing and management [8]. High prevalence rates were found in Denmark (25–88%) and Canada (18–82%) [9, 10]. In Spain, the prevalence found in reared pigs varied from 28.7 to 48.7% [11, 12].

The relevant diffusion of the parasitosis is mainly due to the high fecundity of female *A. suum* worms (up to 200,000 eggs per female per day) and to the strong structure of the eggs, which guarantees their survival in the external environment even up to 4 years [13].

The biological cycle is direct and begins with the ingestion of embryonated eggs containing the infective larva, an early third-stage (L3) larva covered by the cuticle of the second stage larva (L2) [14]. Piglets can become infected shortly after birth by ingesting embrionated eggs smearing the mammary skin of the sow or directly through food and drinking water contaminated with eggs, especially in polluted soil [2]. The eggs hatch in the stomach and small intestine and release the ascarid L3 larvae, which penetrate the intestinal wall and start their hepatic-pulmonary migration, first to the liver via the portal system [15]. In the liver, the larvae lose the cuticle and enter the lungs via the systemic circulation, where they embolize in the pulmonary capillaries, enter the alveoli, are coughed up and then swallowed, reaching the small intestine approximately two weeks after infection [16]. Once back in the small intestine, the larvae moult into the fourth-stage (L4) larva, where most or all of the parasites are expelled from the intestine at 14–21 days post-infection (self-cure). Some of these larvae may remain in the small intestine for the rest of their lives, developing into preadult and then adult worms that reach sexual maturity at 6–10 weeks of age [14].

Earthworms and dung beetles may play a role in the transmission, as they can ingest *Ascaris* eggs while feeding on soil and feces. The eggs can then hatch and infective larvae can remain in the tissues of earthworms and dung beetles until they are eaten by the pig host, acting as potential paratenic hosts in the life cycle of *A. suum* [17, 18].

The migration of larvae within the liver can lead to the onset of multifocal interstitial hepatitis in sensitized animals, characterized by fibrosis appearing as white spots (milk spots) [19, 20], while pulmonary alterations caused by migrating larvae include pneumonia, pleurisy and allergic asthma in infected pigs [21].

Although the course of ascariasis in pigs is usually subclinical, clinical infections can occur in growing pigs [22].

The pathological effects of adult *A. suum* in the small intestine are less dramatic than those of larval migration, but they can cause a chronic malabsorption syndrome with atrophy of the intestinal villi, resulting in reduced weight gain and poor feed conversion [23, 24].

In rare cases, obstruction of the intestinal lumen may happen in heavily infested animals, as well as obstruction of the bile ducts due to the migratory tendency of ascarids, resulting in jaundice [2].

The present study reports an unusual localization of *A.* *suum* worms in the biliary system of a piglet.

## Materials and Methods

In February 2022, a 30-days-old piglet slaughtered in Milis (Latitude: 40° 00′ 49″ N Longitude: 8° 35′ 53″ E) in the Oristano province (Sardinia—Italy) presented *Ascaris* worms in the hepatic biliary tree. The piglet was the seventh of a litter born from a 3-year-old Sarda multiparous sow in which *Ascaris* eggs were found through the copromicroscopical examination. The piglets were raised indoor, in a box with solid concrete flooring together with his mother and the sow before the parturition had access to the paddock outdoor where she could root.

At the time of the home slaughtering, according to the Legislative Decree 2 February 2021 n.27 which provides the inspection in only 10% of the home slaughtering, the official veterinarian did not attend, and the intestine was discarded. The liver was examined, and transverse cuts were performed to allow the isolation of worms within the bile ducts. The worms were washed with 0.9% NaCl solution, and then examined under a stereo microscope, the measurements were taken and their identification was carried out using the morphometric keys available in literature [25]. The DNA was extracted from the middle portion of a collected worm using a G-spin^™^ Total DNA Extraction kit (iNtRON Biotechnology, Korea), according to the manufacturer’s instructions. The rDNA region comprising the ITS-1 and the ITS-2 was amplified by PCR using 100 pmol of primers NC5 and NC2 as described by Zhu *et al*. [26]. PCR products were sent to an external sequencing service (Eurofins Genomics, Ebersberg, Germany) and the sequences obtained were compared with those found in the National Centre for Biotechnology Information (NCBI) database using Basic Local Alignment Search Tool (BLAST) (http:// www. ncbi. nlm. nih. gov/ BLAST/).

Before the parasite removal, the liver was subjected to ex vivo ultrasound examination using a portable ultrasound unit (My Lab Alpha, Esaote, Florence, Italy) equipped with a multifrequency (3–13 MHz) linear probe (SL1543, Esaote, Florence, Italy).

The liver was also processed for the histological examination as follows: tissue samples of the liver were immediately fixed in 10% buffered formalin for 48 h, dehydrated with increasing alcohol concentrations and xylene in an automatic tissue processor and paraffin-embedded. Sections of 3 μm thickness were stained with hematoxylin and eosin (HE) and were observed at light microscopy (Nikon Eclipse 80i). Parasites were categorized according to the available literature [27].

## Results

**Fig.1:**
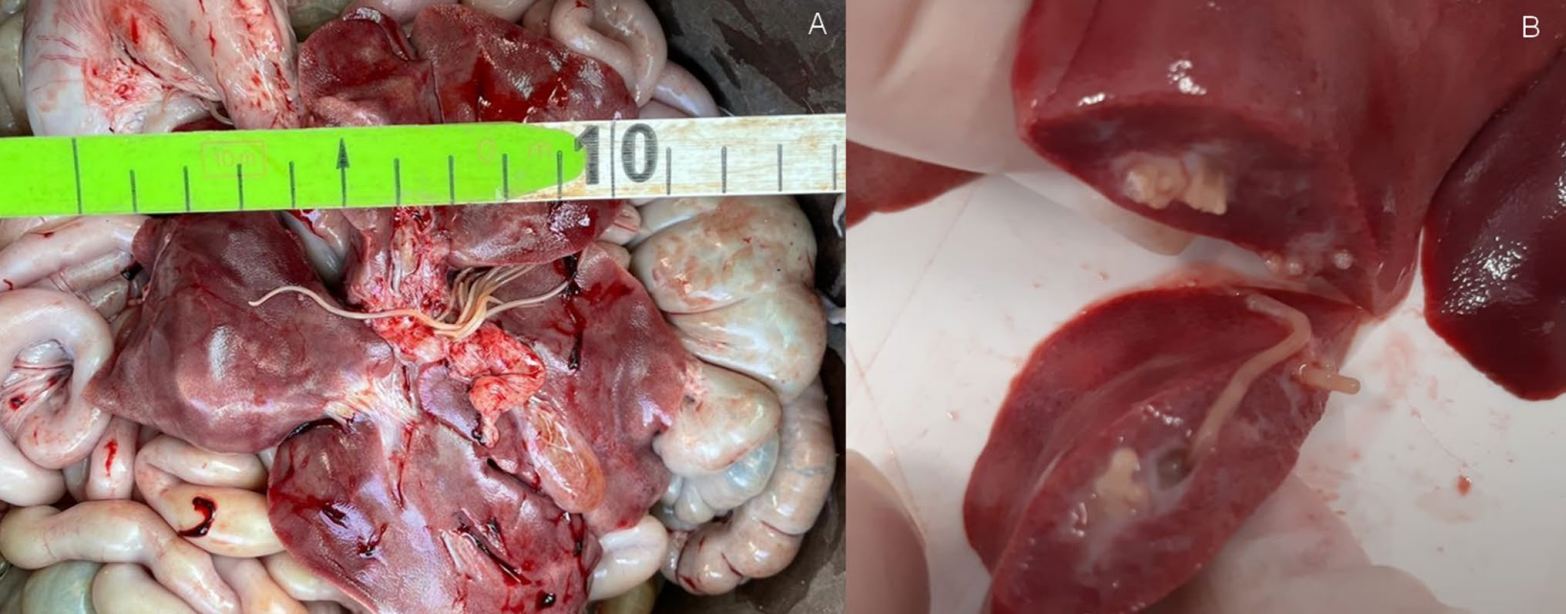
Gross examination of the liver, with several preadult specimens of 7 cm mean length (**A**), and a detail of nematodes occupying the bile ducts (**B**). The scale bar in **A** has centimeter (cm) as unit

The presence of ascarids in the biliary system was detected only in a single piglet within the whole litter. A total of 43 preadult nematodes, with a mean length of 8 cm and a mean width of 1.7 mm, were found in the gallbladder and biliary tree (Fig. 1).

Parasites were morphologically identified under the stereomicroscope as belonging to the genus *Ascaris*, presenting the characteristic three lips, without interlabia and cervical alae*,* as described in the literature with morphological keys [25].

Molecular biology allowed to identify the specimens as *A.* *suum*, and the nucleotide sequence obtained had a 99% similarity with a sequence from Thailand (accession number MF358937.1) deposited by Sadaow *et al*. [28].

**Fig.2:**
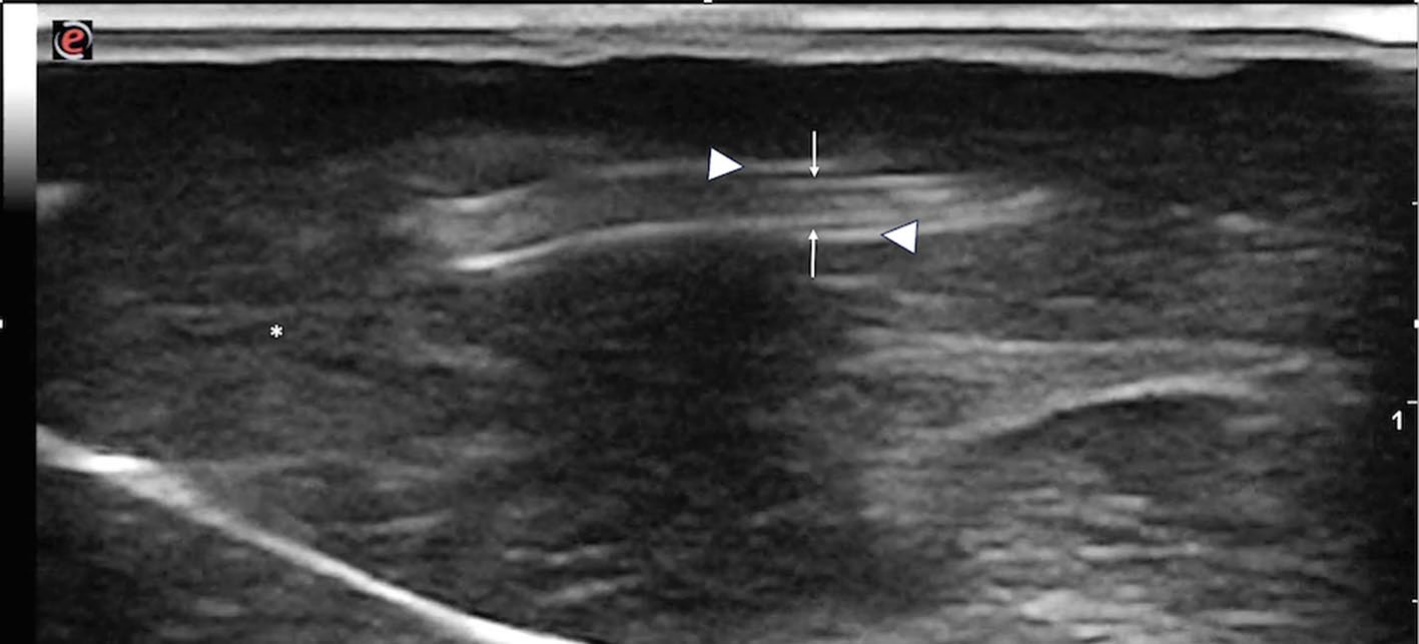
Ex vivo ultrasound image of the liver. The white arrows indicate a longitudinal parasite section, white arrowheads indicate the thickened walls of the bile ducts. *Liver parenchyma

The hepatic ultrasound examination performed before the isolation of the parasites revealed the presence of several nematodes in the lumen of dilated and thick-walled bile ducts (Fig. 2). In longitudinal section, worms appeared as nonshadowing linear, tubular structures, characterized by two parallel hyperechoic lines separated by a hypoechoic zone. In transverse section, the worms had a “doughnut-like” appearance with a circular hyperechoic nonshadowing wall and a hypoechoic center.

**Fig.3:**
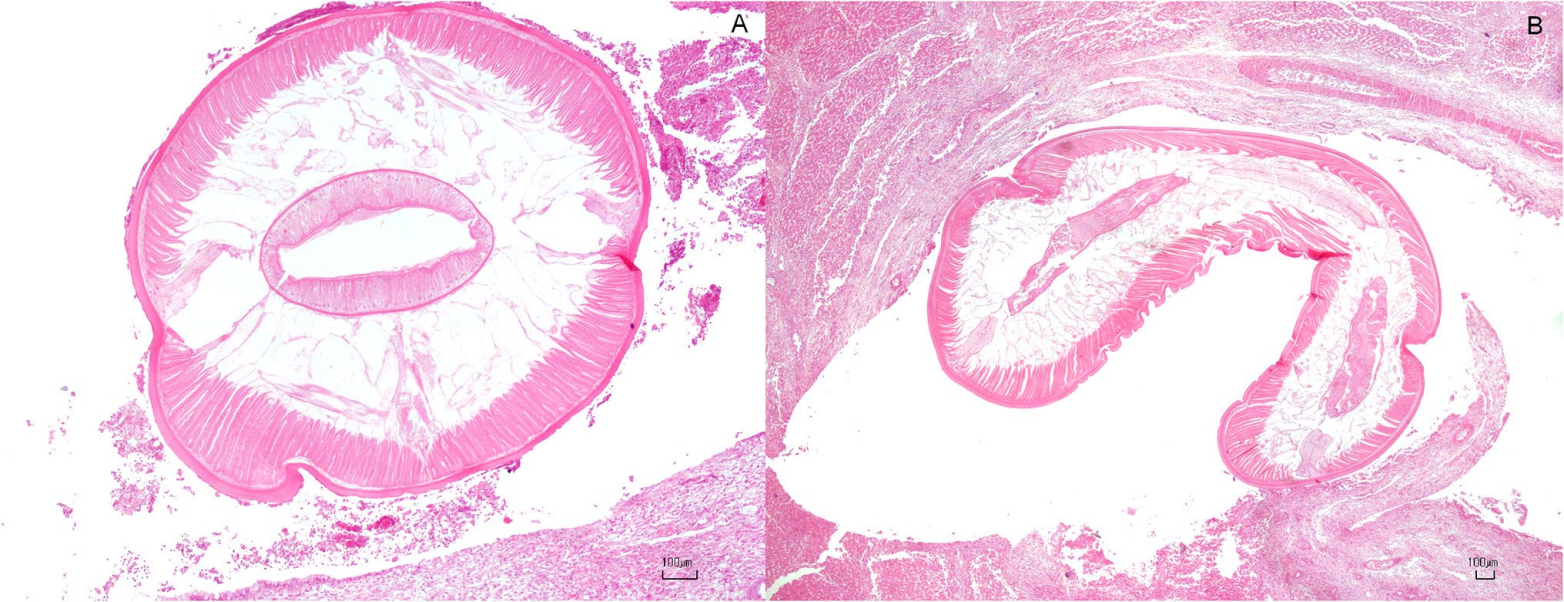
Transverse (**A**) and longitudinal (**B**) histological sections of nematodes with a smooth cuticle, coelomyarian musculature, intestinal tract lined by columnar uninucleated cells within a pseudocoelom. H.E bar 100 µm

Macroscopically, the liver was moderately enlarged, with accentuated lobular pattern and scattered pales areas admixed with multifocal hemorrhages. Multifocally, the parasitized bile ducts were severely enlarged. The expansion of portal areas determined the compression of adjacent sinusoids and hepatic cords with a severe, chronic inflammatory infiltrate around the bile ducts. The latter were up to 5 mm in diameter, lined by hyperplastic epithelium (biliary epithelial hyperplasia) and filled by sections of nematodes. They were also surrounded by a moderate number of plump reactive fibroblasts and lesser numbers of fibrocytes. The cells were observed embedded in a moderate amount of eosinophilic fibrillary material (collagen) interpreted as fibroplasia, often infiltrated by a high number of eosinophils, fewer lymphocytes, macrophages and plasma cells. Within the lumen of bile ducts, sloughed epithelial cells and cellular debris were admixed with longitudinal up to 4 mm and transverse (up to 1.5 mm) sections of nematodes with a smooth cuticle, coelomyarian musculature, intestinal tract lined by columnar uninucleated cells within a pseudocoelom (Fig. 3).

Adjacent to bands of fibrosis, there were limitedly shrunken and hypereosinophilic individualized hepatocytes (atrophy). In less affected areas, portal areas were multifocally expanded by fibrous connective tissue that often connects adjacent portal areas (bridging fibrosis), admixed with increased numbers of small bile ducts (ductular reaction) and previously described inflammatory cells. The parasites were histologically identified as belonging to the Family Ascarididae, based on the microstructural features indicated by the literature [27].

## Discussion

Here, we present a rare case with preadult worms of *A. suum* in the biliary system of a piglet. To our knowledge, this is the second literature report of this localization of *A. suum* in a piglet. In contrast to humans, where hepatobiliary ascariasis is a known complication of *A. lumbricoides* infection, the only other report of hepatobiliary *A. suum* infection in pigs on a global scale dates back 70 years [29].

There are only a few reports of ascarid migration into host bile ducts in the literature, one by *Toxocara vitulorum* in a buffalo [30] and a more recent report of adult *Parascaris* sp. in the biliary tree of a foal [31].

In addition, there are a few reports of accidental infection of *A.* *suum* in hosts other than pigs, such as cattle [32], sheep [33] and a dog [34], but with the exception of the sheep case, only larvae were found in these unusual hosts.

Unfortunately, due to the absence of gut, it was not possible to define the pathogenesis of the infection. However, as has been reported with *A.* *lumbricoides* infection in humans, the *A. suum* worms likely migrated from the intestine through the ductus choledocus to the liver [35]. Biliary ascariasis in humans is a rare disease in nonendemic areas, but in endemic areas it is the cause of a significant proportion of biliary and pancreatic diseases. In particular, in India, in the highly endemic region of Kashmir, ascariasis was found to be the cause in 36.7% of 109 patients with confirmed biliary and pancreatic diseases [35]. In the human host, altered intestinal motility is the usual condition for ascarids to reach the duodenum from their natural habitat, the jejunum. This may be due to a severe worm infection or other intestinal infections of viral, bacterial, or parasitic origin, but the host response to an adult worm may also alter vasomotor reflexes and secretory responses, which in turn affect intestinal tone and motility [36]. In this condition, worms can reach the Vater ampulla from the duodenum and then they can be located: (a) in the ampulla itself, (b) in the common bile duct, or (c) in the hepatic ducts or somewhere in the biliary tree [1].

It has been reported that worms in humans actively migrate in and out of the bile ducts from the duodenum and therefore can lead to even transient biliary tree parasitosis [35].

The number of parasites found in the bile ducts and the nature of the histologic reaction around them, seems to rule out the hypothesis of backward migration into the small intestine. Unfortunately, it was not possible to examine the small intestine of the piglet reported here, so it is impossible to know whether there was a concomitant intestinal infection. It would be interesting to follow the infection pathway to assess whether the parasites could reach sexual maturity, mate and complete the life cycle in the biliary system. Such an experiment could have been performed by *in vivo* ultrasound examination, which would have made it possible to exclude a possible concomitant intestinal infection and to detect ascarids infection at the level of the bile ducts. The ultrasonographic appearance of nematodes has been previously described in humans and animals [37–39]. In longitudinal section, they appear as linear “train track” structures consisting of two parallel hyperechoic lines representing the cuticle, separated by a narrow hypo/anechoic line representing the alimentary canal. In cross-section, they have a “donut” appearance characterized by a hyperechoic circular wall with a hypoanechoic centre. The sonographic diagnosis of biliary ascariasis has been prevoiusly described in human medicine [37, 40]. However, to our knowledge, this report is the first to describe the ultrasonographic appearance of roundworms in the biliary system of pigs, although the examination was performed on an organ and not on a live animal. Because roundworms are easily detected by ultrasound, this test could provide an *in vivo* diagnostic method for detecting parasites in the biliary tract of piglets.

Given the unusual nature of this finding, it is difficult to identify predisposing factors for this *A. suum* localization, such as factors related to age, breed, concurrent intestinal infections, diet, etc.

Although the presence of the preadult worms was a slaughter finding with no associated pathology, it suggests that ascariasis should be considered in the differential diagnosis of pigs with hepatobiliary disease.

## Data Availability

All the data and materials related to the current study are available from the corresponding author on reasonable request.
